# Clinical features and surgical treatment of sacral schwannomas

**DOI:** 10.18632/oncotarget.16968

**Published:** 2017-04-08

**Authors:** Weibo Pan, Zhan Wang, Nong Lin, Xin Huang, Meng Liu, Xiaobo Yan, Zhaoming Ye

**Affiliations:** ^1^ Department of Orthopaedics, The Second Affiliated Hospital of Zhejiang University School of Medicine, Centre for Orthopaedic Research, Orthopedics Research Institute of Zhejiang University, Hangzhou, China

**Keywords:** sacral schwannomas, clinical features, intralesional excision

## Abstract

**Background:**

Sacral schwannoma is relatively rare and both the early diagnosis and appropriate treatment of sacral schwannomas are equally very challenging.

**Methods:**

11 sacral schwannoma cases operated at the Second Affiliated Hospital, School of Medicine, Zhejiang University, from 2012 to 2016, were investigated retrospectively and 10 were followed up. All patients were assessed with X-ray, CT and MRI, and underwent an intralesional excision.

**Results:**

One patient was male, nine were female and the average age was 48 (ranging between 31 and 63). Three patients suffered from back and leg pain, and seven had no obvious symptoms. The average blood loss during surgery was 980ml (ranging between 100 and 2,000ml). Six patients underwent preoperative biopsy. The surgeries were performed *via* the combination of an anterior and posterior approach in two patients, a posterior approach in seven patients, and an anterior approach in one patient. Residual tumors were not detected in all patients after surgery. Unfortunately, the postoperative complications occurred in three patients, namely bowel and bladder dysfunction (two patients) and cerebrospinal fluid leakage with secondary intracranial infection (one patient). The average follow-up was 22.7 months (8-44 months). All patients were relieved from preoperative symptoms after the last follow-up.

**Conclusions:**

The typical findings of our cases in MRI were a well-circumscribed lesion with a heterogenous signal intensity on T2-weighted image, which may be helpful for preoperative decision-making. Intralesional excision can be successfully performed using single anterior or single posterior or both, and is an important procedure in the treatment of sacral schwannomas.

## INTRODUCTION

Schwannomas are mostly benign neurogenic tumors arising from Schwann cells of the peripheral nerve sheath [1]. Spinal schwannoma are relatively common, accounting for 25% of all primary spinal tumors [2]. However, sacral schwannomas are less common and reported less than 1% to 5% of all spinal schwannomas [3]. CT and MRI are very important for the diagnosis of sacral schwannomas. Many patients do not notice pain or numbness until the tumor spread into adjacent spaces and become larger [4, 5]. Therefore, the tumor volume is often quite large at the first time of diagnosis. Due to the complex anatomical structure of sacrum, surgical treatment of such tumors is often challenging. Additionally, a variety of complication can be expected after surgery [6]. Therefore, both the early diagnosis and appropriate treatment of sacral schwannomas are equally very important.

Here we present 10 cases of patients with sacral schwannomas and discuss the clinical features and the surgical strategy of sacral schwannomas.

## RESULTS

One patients were male, nine were female and the average age was 48 (ranging between 31 and 63). Three patients suffered from back and leg pain at the time of consultation. Seven patients with vague symptoms or symptomless were found incidentally. Six patients underwent preoperative biopsy and histological analysis and four patients had no such analysis. More data can be seen in Table 1.

**Table 1:** Clinical summary of the sacral schwannomas

Patient No.	Sex/Age	Preoperative biopsy	Blood loss(ml)	Size(mm)	Procedure	Complications	Follow-up time (month)
1	F/46	No	100	60*30	Anterior approach	None	24
2	F/41	No	800	80*60	Posterior approach	None	44
3	M/57	Yes	2000	77*75	Posterior approach	Bowel and bladder dysfunction	40
4	F/43	No	800	43*67	Anterior and posterior approach	None	26
5	F/57	No	200	58*99	Anterior and posterior approach	None	25
6	F/31	Yes	1400	68*38	Posterior approach	Cerebrospinal fluid leakage with secondary intracranial infection	20
7	F/46	Yes	1600	57*62	Posterior approach	None	18
8	F/63	Yes	1500	67*79	Posterior approach	None	13
9	F/37	Yes	400	35*31	Posterior approach	None	9
10	F/59	Yes	1000	67*39	Posterior approach	Bowel and bladder dysfunction	8

**Figure 1:**
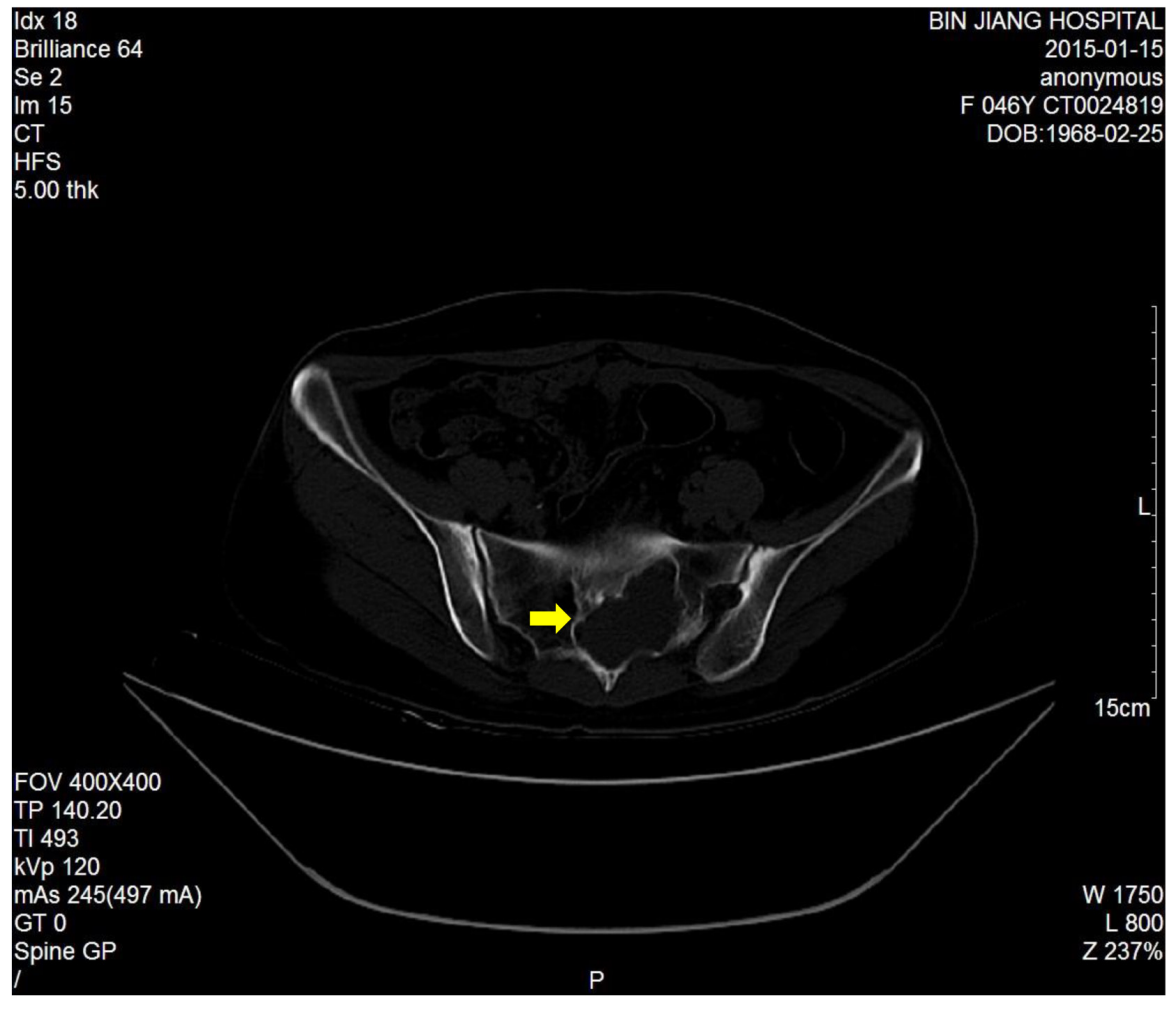
Axial CT scan showed a huge left sacral expansive lesions with marginal sclerosis(S1-2)

**Figure 2:**
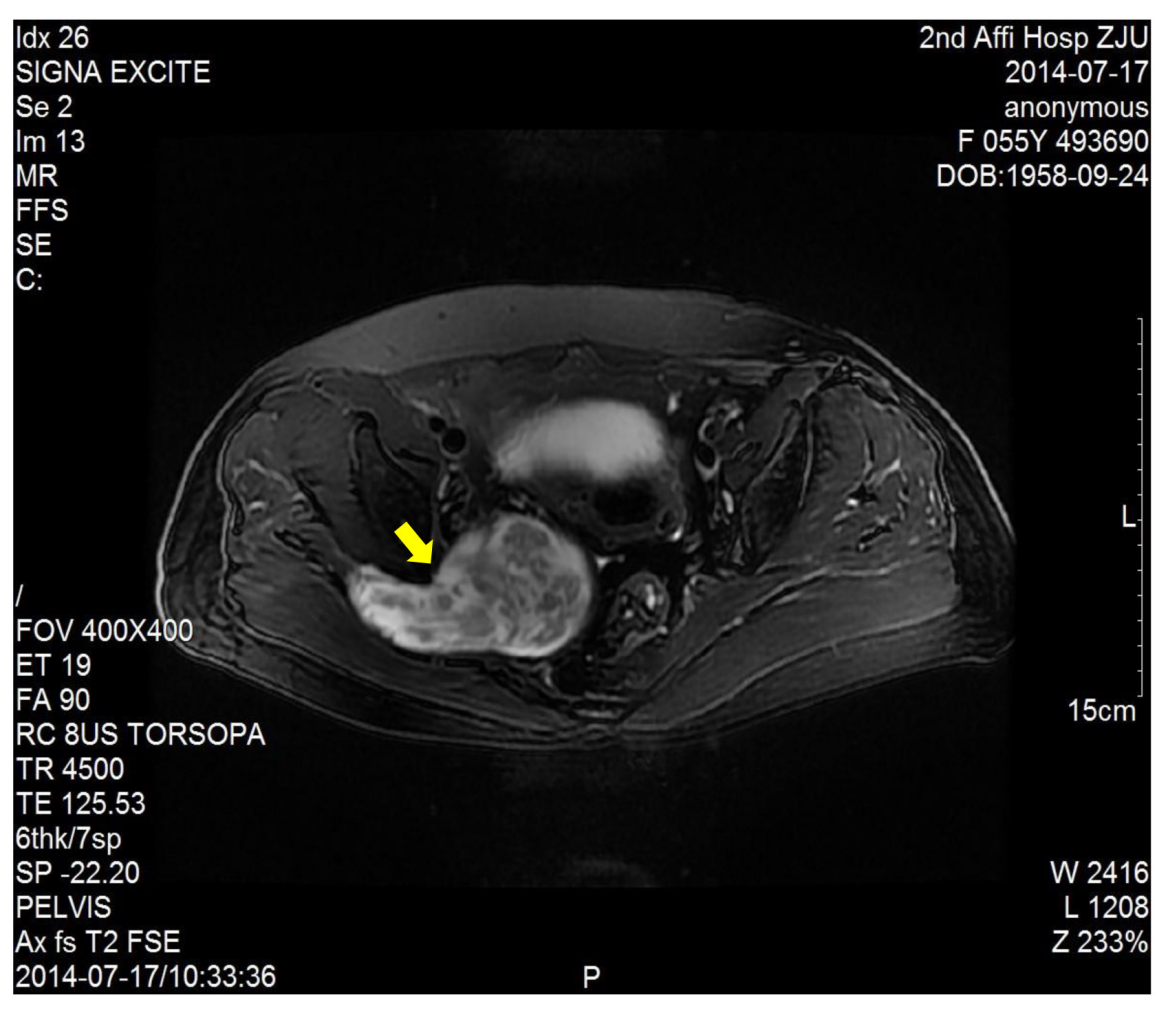
T2-weighted image showed a right sacral mass as a dumbbell-shaped configuration and mixed low signal intensity on the basis of high signal intensity

**Figure 3:**
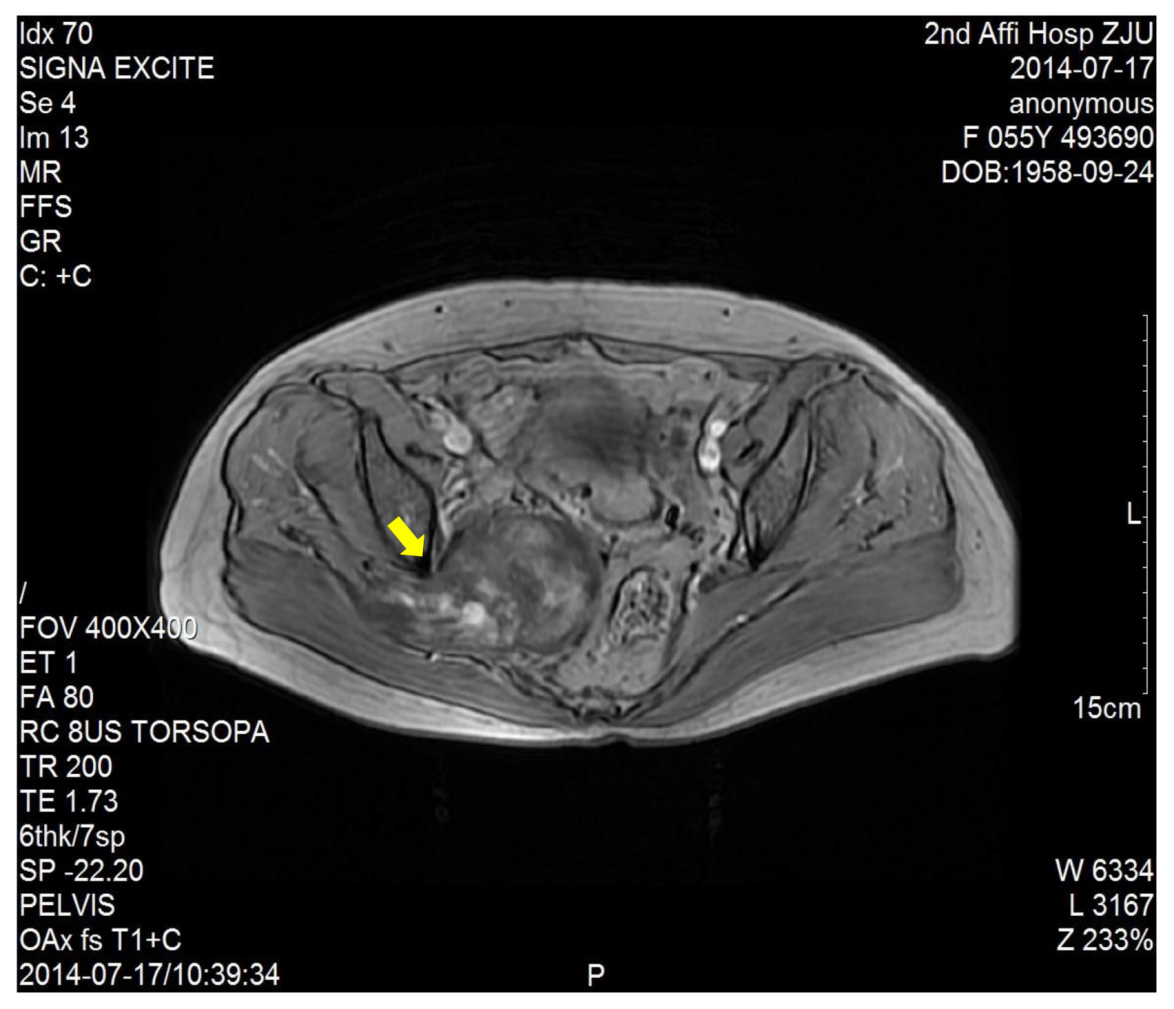
T1-weighted image showed a right sacral mass as a dumbbell-shaped configuration and mixed high signal intensity on the basis of low signal intensity

**Figure 4:**
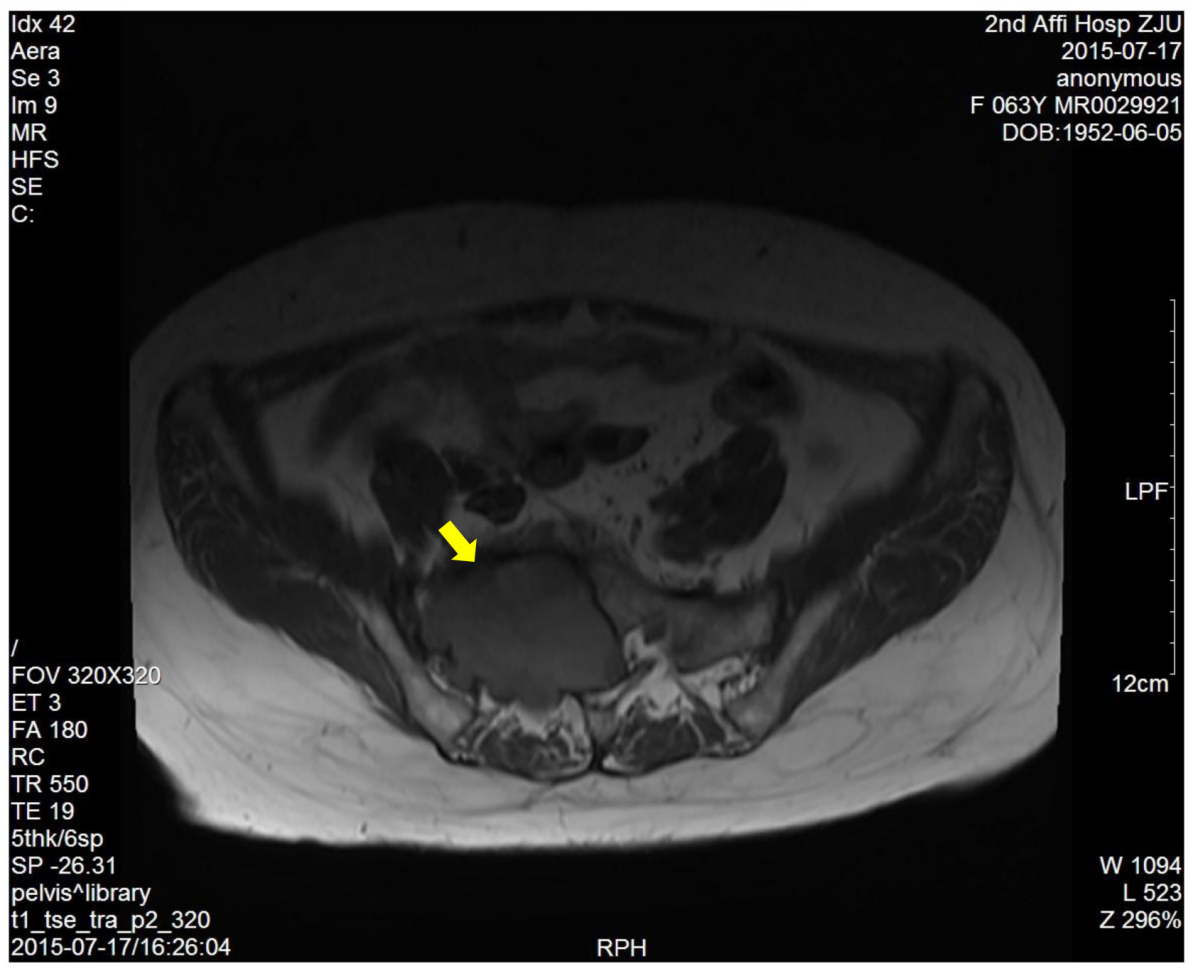
T1-weighted image showed a right sacral mass with low signal intensity

**Figure 5:**
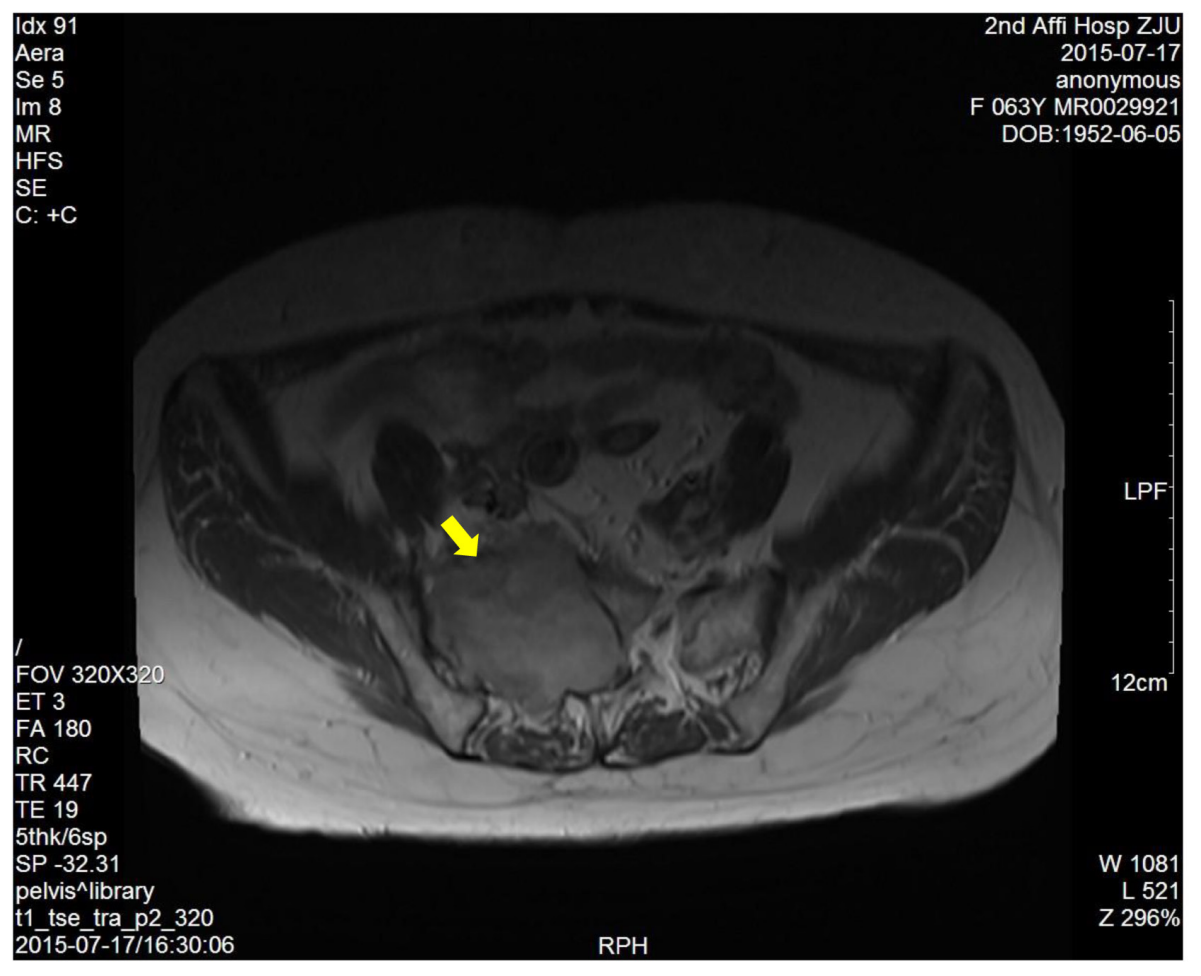
The enhanced T1-weighted image showed a right sacral mass with uneven enhancement

The dimension of the tumors was also shown in Table 1. Lumbosacral CT of our cases were characterized by expansive lesions located in high-sacra such as sacrum 1 and 2, mass appearance on the lateral caudal, well-circumscribed lesions with marginal sclerosis, and the overall benign presentation (Figure 1). In our study, the typical findings of sacral schwannomas in MRI were a well-circumscribed lesion with a heterogenous signal intensity on T2-weighted image. On T2-weighted image, low signal intensity was mixed on the basis of high signal intensity, which was found in our all cases (Figure 2). On T1-weighted image, slightly high signal intensity was mixed on the basis of low signal intensity, which was shown in two cases (Figure 3). Low signal on T1-weighted image was shown in eight patients (Figure 4). T1-weighted images of all cases are characterized by heterogenous enhancement (Figure 5). Average blood loss during surgery was 980ml (ranging between 100 and 2,000ml). Seven patients had only posterior approach as their tumors were limited to the sacrum; two patients went through both anterior and posterior interventions as their tumors extended to the anterior and posterior bone limits of the sacrum; one had just anterior intervention because the tumor was localized to the presacral region. All patients underwent an intralesional resection (piecemeal subtotal excision or shaving) to identify and preserve sacral nerve roots as much as possible. All patients were performed histological examinations after surgery to confirm the diagnosis of sacral schwannomas. Two patients had postoperative bowel and bladder dysfunction after surgery. But they gradually recovered after four months and three weeks, respectively. One patient had cerebrospinal fluid leakage and secondary intracranial infection. This patient finally recovered after several lumbar drainage and anti-infection treatment. All patients were ambulatory postoperatively.

Average follow-up period is 22.7months (8-44 months). All patients were assessed with MRI every time. At the last follow-up there were no residual or recurrent symptoms at the clinical or radiological findings in all patients.

Many common features of sacral schwannomas have been revealed in our study, including female dominancy, middle age, local invasive nature together with vertebral body erosion and large size but benign histology, mild clinical presentation, common preoperative misdiagnosis and good prognosis after intralesional excision. Additionally, the typical MRI findings of sacral schwannomas in our cases were a well-circumscribed lesion with a heterogenous signal intensity on T2-weighted image.

## DISCUSSION

Sacral schwannomas is usually found incidentally or when patients present with pain and neurological symptoms such as lower back pain, numbness or paresthesias. In our cases, three patients who presented with back and leg pain then gradually presented with radiculopathy of one or more sacral roots. The other seven patients presented with vague symptoms. Many studies [7–9] showed female dominancy and most often in their 2nd to 5th decades of life in their series, which were consistent with our results (female/male, 9/1; age, ranging between 31 and 63). The early diagnosis of sacral schwannomas is difficult because of patients’ vague aches and minimal symptoms. Moreover, the tumors tend to reach an enormous size and expand towards the anterior, the posterior, pelvis, or in combination due to the relative mobility of the roots and the wide diameter of the spinal canal surrounding them, which makes them usually difficult to manage.

CT and MRI are the most useful imaging modality for preoperative diagnosis of this tumor. Lumbosacral CT of our cases is characterized by lesions centered on the sacral foramen, dumbbell-shaped configuration, mass appearance both inside and outside vertebral canal, widening sacral foramen, well-circumscribed lesions, and the overall benign presentation. Pelvic MRI can provide information about the size, exact localization, and invasion of and relationship with other vital organs. Typical MRI findings of schwannomas have been reported as masses of low signal intensity on T1-weighted images and high signal intensity on T2-weighted images [10, 11]. But in our study, the MRI findings of all sacral schwannomas are a well-circumscribed lesion with complex signal intensities on T2WI. This heterogeneous appearance may be due to remote hemorrhage and degeneration, necrosis and liquefaction of tumor tissues. So, a well-circumscribed lesion with a heterogenous signal intensity on T2-weighted image may be more helpful for preoperative diagnosis.

In this study, the biopsy was performed in six patients and no complication like infection or hemorrhage was seen. But this was not performed in four featured cases given the benign-appearing features of the tumor. Although the procedure has a potential risk of complications such as infection, site-related problems, wound closure issues, or hemorrhage [12–15], it is helpful for the predetermination of the nature of the tumor particularly when the diagnosis of sacral schwannomas is uncertain due to its atypical symptoms and imaging findings. As we all know, misdiagnosis of the disease will gradually promote tumor growth, making the operating procedure more difficult.

Although surgical resection of the sacral schwannoma is complex because of its anatomic location and propensity for local recurrence, it is recommended for management of spinal schwannomas. Given the extent of the tumor, and the anatomical relationships, different types of approaches were performed in our study. When the tumor is confined to the front of the sacrum, anterior approach is preferred. Otherwise, most of these tumors can be removed *via* posterior approach alone. Additionally, posterior approaches can also be performed to remove sacral schwannomas with large presacral component *via* proper fenestration. But when extraspinal portion is greater than the intraspinal and vertebral body portions or erosion of lumbar vertebral body, performing posterior approach alone cannot achieve complete resection and thus a combined approach is preferred. For example, one patient in our study underwent an anterior and posterior approach because CT revealed a destructive and expansile mass invasion of the spinal canal and erosion of L5 vertebral body.

Given that sacral schwannoma is a benign tumor, sacral nerve roots should be preserved as much as possible. Pongsthorn et al [7] prefer the piecemeal subtotal excision for giant sacral schwannoma to avoid unnecessary neurological deficit, and achieve a good outcome. Togral et al [16] think the frequently preferred treatment for sacral schwannomas is piecemeal subtotal excision due to its rare local recurrence and transformation to malignancy. Çağlı et al [6] think a simple intralesionary excision is an appropriate choice for giant sacral schwannomas. All of our patients were performed intralesional resection (piecemeal subtotal excision) and the prognosis is good after total tumor removal on the whole. Although two cases experienced nerve dysfunction after operation due to thinner affected nerve roots, they gradually restored function over a short period of time. Pongsthorn et al [7] reported a recurrence of 16%. Çağlı et al [6] detected a small residual tumor in two patients but no recurrence. No recurrence was found in our patients treated with this strategy. Perhaps the follow-up time (mean 1.9 years) was relatively shorter. Additionally, some authors prefer aggressive tumor management for sacral schwannoma *via* wide resection and total sacrectomy given the risk of local recurrence [17–19]. But this debated strategy often results in sacral nerve dysfunction and more blood loss. Sahakitrungruang et al [19] think en bloc sacrectomy can be safely conducted for sacral tumors and found no recurrence. While Yu [20] reported a recurrence of 40%(2/5) with gross total resection for sacral schwannoma. The recurrence of spinal schwannoma tended to occur more often in younger patients and in the lumbar segment. The pre-operative size of the spinal schwannoma and intralesional resection are the main risk factors for the local recurrence [21]. However, the risk factors for local recurrence of sacral schwannoma has not been studied. Sacral tumor resection with laparoscopic technique is increasingly performed. But this technique remains in its infancy and requires further improvement [22].

Although schwannoma is usually not rich in blood supply, there was relatively more blood loss during intralesional excision in our study (mean 980ml, ranging between 100 and 2,000ml). Pongsthorn et al [7] reported the intraoperative blood loss was 2572 g (range 483-5301g). Intraoperative hemorrhage mainly results from venous plexus of sacral canal, instead of the tumor itself. Managing the venous plexus hemorrhage can effectively decrease the intraoperative hemorrhage and keep the operating field clear.

## CONCLUSIONS

The typical findings of our cases in MRI were a well-circumscribed lesion with a heterogenous signal intensity on T2-weighted image, which may be helpful for preoperative decision-making. Intralesional excision can be successfully performed using single anterior or single posterior or combined approach, and is an important procedure in the treatment of sacral schwannomas.

## MATERIALS AND METHODS

Between December 2012 and September 2016, 11 sacral schwannoma patients were operated at Department of Orthopaedic Surgery, the Second Affiliated Hospital, School of Medicine, Zhejiang University. Finally, 10 were fully followed up and one was lost to follow-up.

All patients were assessed with plain radiography, computerized tomography (CT) and magnetic resonance imaging (MRI). T1-weighted MRI using the contrast agent gadopentetate dimeglumine was performed on all patients. The preoperative diagnosis of sacral schwannoma was suspected on the basis of the clinical history and presentation and imaging studies. It was finally confirmed by the pathological results. Radiograph of pelvic cannot clearly show sacral lesions. CT and MRI can clearly show the lesion of sacral schwannoma and the relationship between the mass and other vital organs. A lytic defect with sclerotic margins of the sacrum is usually detected on X-ray and CT [16, 23]. Lumbosacral CT of typical cases are often characterized by lesions centered on the sacral foramen, dumbbell-shaped configuration, mass appearance both inside and outside vertebral canal, widening sacral foramen, well-circumscribed lesions, and the overall benign presentation. Typical findings of schwannomas in MRI are low signal on T1-weighted and high signal on T2-weighted images [10]. In some cases, preoperative biopsy was performed to obtain histologic proof of the lesion. But this was not performed in our featured cases given the benign-appearing features of the tumor.

Patient No.1 with a tumor involving the left S1 body and presacral region was operated *via* anterior approach (left iliofemoral approach). Patient No.4 with a presacral mass lesion involving the L5 vertebral body went through both anterior and posterior interventions. Tumor removal was performed by the combination of an anterior and posterior approach in Patient No.5 because the giant sacral schwannoma was located in sciatic macroporous and across the pelvis. The rest of the patients (7/10) were operated *via* posterior approach.
